# Controlling Video Stimuli in Sign Language and Gesture Research: The *OpenPoseR* Package for Analyzing *OpenPose* Motion-Tracking Data in *R*

**DOI:** 10.3389/fpsyg.2021.628728

**Published:** 2021-02-19

**Authors:** Patrick C. Trettenbrein, Emiliano Zaccarella

**Affiliations:** ^1^Department of Neuropsychology, Max Planck Institute for Human Cognitive and Brain Sciences, Leipzig, Germany; ^2^International Max Planck Research School on Neuroscience of Communication: Structure, Function, and Plasticity (IMPRS NeuroCom), Leipzig, Germany

**Keywords:** *R*, linguistics, psychology, neuroscience, sign language, gesture, video stimuli

## Abstract

Researchers in the fields of sign language and gesture studies frequently present their participants with video stimuli showing actors performing linguistic signs or co-speech gestures. Up to now, such video stimuli have been mostly controlled only for some of the technical aspects of the video material (e.g., duration of clips, encoding, framerate, etc.), leaving open the possibility that systematic differences in video stimulus materials may be concealed in the actual motion properties of the actor’s movements. Computer vision methods such as *OpenPose* enable the fitting of body-pose models to the consecutive frames of a video clip and thereby make it possible to recover the movements performed by the actor in a particular video clip without the use of a point-based or markerless motion-tracking system during recording. The *OpenPoseR* package provides a straightforward and reproducible way of working with these body-pose model data extracted from video clips using *OpenPose*, allowing researchers in the fields of sign language and gesture studies to quantify the amount of motion (velocity and acceleration) pertaining only to the movements performed by the actor in a video clip. These quantitative measures can be used for controlling differences in the movements of an actor in stimulus video clips or, for example, between different conditions of an experiment. In addition, the package also provides a set of functions for generating plots for data visualization, as well as an easy-to-use way of automatically extracting metadata (e.g., duration, framerate, etc.) from large sets of video files.

## Introduction

Researchers in linguistics, psychology, and neuroscience who are studying sign language and gesture frequently present their participants with pre-recorded video stimuli showing actors performing manual gestures. Such gestures may be lexicalized signs of a natural sign language which can be combined to build up complex meanings (Klima et al., 1979; Mathur and Rathmann, 2014; Cecchetto, 2017) and are primarily processed by the brain’s left-hemispheric core language network (Emmorey, 2015; Trettenbrein et al., 2021). Alternatively, non-signers may use a wide variety of so-called co-speech gestures to support different communicative functions in situations where gestures are produced spontaneously alongside speech (McNeill, 1985; Krauss, 1998; Özyürek, 2018).

The reliance on video clips as stimulus materials presents researchers in sign language and gesture studies with a number of challenges. While controlling for primarily technical aspects of the video material (e.g., duration of clips, encoding, framerate, etc.) is rather straightforward, it is strikingly more difficult to capture aspects of the video material which are related to the actor and the specific movements they performed. For example, while the length of video clips in an experiment may be perfectly matched across the different conditions, systematic differences could nevertheless exist with regard to the speed, duration, and extent of the movements performed by the actor in each condition. In a hypothetical neuroimaging experiment on sign language, such systematic differences could for example, lead to unexpected response of parts of cortex that are sensitive to biological motion across conditions, thus distorting the actual contrasts of interest. By quantifying the bodily movements of the actor in a video clip it becomes possible to control for potential differences in these movement patterns across different conditions, or use information about velocity or acceleration as further regressors in a statistical model.

Up to now, researchers have usually focused on controlling their video stimuli only with respect to certain technical properties. Some have used device-based optic marker motion-tracking systems, but only to create stimulus materials which displayed gestures in the form of point-light displays instead of a human actor (Poizner et al., 1981; Campbell et al., 2011). A number of markerless motion-tracking systems (e.g., Microsoft Kinect; Zhang, 2012) and tools for analyzing these data exist (Trujillo et al., 2019), but using them for creating video stimuli with ultimately only two dimensions in many cases may constitute too big of an expenditure in terms of time, effort, and hardware requirements. Also, neither optic marker nor markerless motion-tracking systems can be retroactively applied to videos already recorded. As a result, the possibility that systematic differences in video stimulus materials may be concealed in the actual motion properties of the actor’s movements has at times been disregarded in sign language and gesture research.

Recent technical advances in video-based tracking systems offer an exciting opportunity for creating means to control video stimuli that go beyond technical aspects such as clip duration by recovering different parameters (e.g., velocity or acceleration) of the actor’s movements performed in the video. A number of existing tools may be used to quantify motion using pixel-based frame-differencing methods (e.g., Paxton and Dale, 2013; Kleinbub and Ramseyer, 2020; Ramseyer, 2020), but they tend require a static camera position, very stable lighting conditions, and do not allow for the actor to change their location on the screen. In contrast, computer vision methods such as *OpenPose* (Cao et al., 2017, 2019) deploy machine learning methods which enable the fitting of a variety of different body-pose models to the consecutive frames of a video clip. By extracting body-pose information in this automated and model-based fashion, such machine learning-based methods essentially make it possible to recover the position of the actor’s body or parts of their body (e.g., head and hands) in every frame of a video clip. Based on this information contained in the body-pose model, the movements performed by the actor in a particular video clip can be recovered in a two-dimensional space without the use of a point-based or markerless motion-tracking system during recording. Consequently, such video-based tracking methods do not require any special equipment and can also be applied retroactively to materials which have been recorded without the aid of motion-tracking systems.

## Methods

**FIGURE 1:**
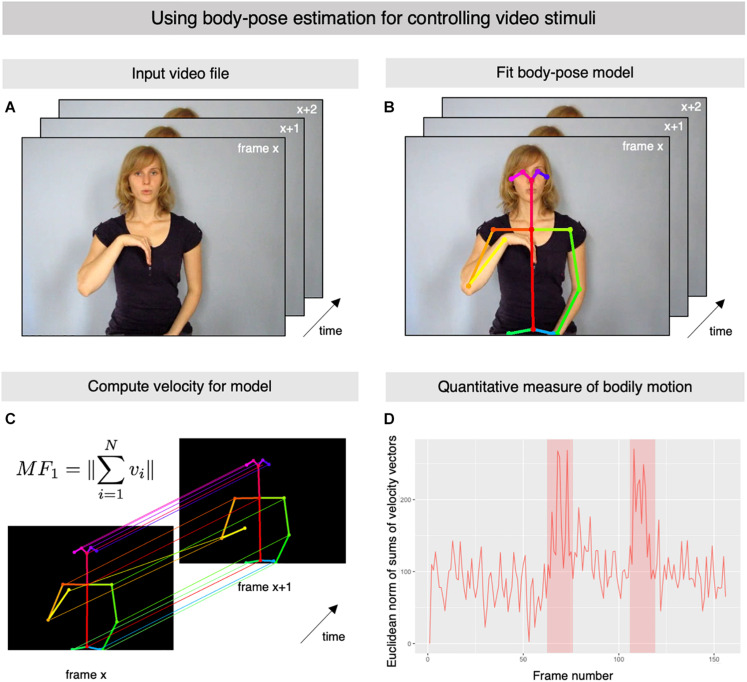
Using body-pose estimation for controlling video stimuli. **(A)** Representative frames of an example video file showing an actor produce the German Sign Language sign for “psychology” (video courtesy of Henrike Maria Falke, gebaerdenlernen.de; license: CC BY-NC-SA 3.0). **(B)** Representative frames from the example input video file illustrating the body-pose model which was fit automatically using *OpenPose*. **(C)** The information from the fit body-pose model is then used by *OpenPoseR* to compute the vertical (y-axis) and horizontal (x-axis) velocity of the different points of the model. Based on these calculations, the software then computes the Euclidean norm of the sums of the velocity vectors. **(D)** The illustrated procedure makes it possible to quantify the total amount of bodily motion in the video using a single measure. Onset and offset the sign are clearly visible as peaks in the plot (shaded areas).

Here we present *OpenPoseR*, a package of specialized functions for the *R* statistics language and environment (R Core Team, 2019) which provides a straightforward and reproducible way of working with body-pose model data extracted from video clips using *OpenPose*. The source code for *OpenPoseR* is freely available from the project’s GitHub page.^1^ In essence, *OpenPoseR* allows researchers in the fields of sign language and gesture studies to quantify the amount of motion pertaining only to the movements performed by the actor in a video clip (Figure 1A) by using information from body-pose models fit by *OpenPose* (Figure 1B). In its current version, *OpenPoseR* provides quantitative measures of motion based on velocity and acceleration of the actor in the video which can be used for controlling differences in these movement parameters, for example, between different conditions of an experiment. More precisely, the package makes it possible to straightforwardly compute the Euclidean norms of sums of all velocity or acceleration vectors (Figure 1C) and thereby provide a quantitative measure of motion for an entire video clip. *OpenPoseR* has already successfully been used to automatically detect onset and offset for a set of more than 300 signs from German Sign Language (Trettenbrein et al., in press). The onset and offset of the sign “psychology” are also clearly visible in the time series depicted Figure 1D. Moreover, an approach similar to the one presented here has shown the general validity of *OpenPose* data (Pouw et al., 2020). In addition to its core functionality the package also provides some functions for generating basic plots for illustration purposes, as well as an easy-to-use way of automatically extracting metadata (e.g., duration, framerate, etc.) from large sets of video files. See Table 1 for an overview of all functions included in the current version. Due to its integration into the larger *R* environment, *OpenPoseR* supports reproducible workflows (e.g., using *R Markdown*; Allaire et al., 2020) and allows for seamless interaction with other *R* packages for further statistical analysis of movement parameters and technical properties of the video materials analyzed. Hence, results of *OpenPoseR* analyses can readily be integrated with other data such as, for example, manual annotation data created using *ELAN* (Lausberg and Sloetjes, 2009).

**TABLE 1:** List of functions available in *OpenPoseR*.

Function	Description
acceleration_x	Computes acceleration for points on x-axis for a given data frame in *OpenPoseR* format.
acceleration_y	Computes acceleration for points on y-axis for a given data frame in *OpenPoseR* format.
clean_data	Discards points with probabilities below a pre-defined cut-off as well as zero values by imposing data.
create_csv	Creates a CSV file in *OpenPoseR* format from raw *OpenPose* JSON output.
det_hand	Determines whether the left, right, or both arms were moved by the actor.
en_acceleration	Computes Euclidean norm of sums of acceleration vectors (x,y).
en_velocity	Computes Euclidean norm of sums of velocity vectors (x,y).
file_acceleration	Computes acceleration for a CSV file generated by create_csv(), using acceleration_x() and acceleration_y().
file_clean	Cleans a CSV file generated by create_csv() using clean_data().
file_en_acceleration	Computes en_acceleration() for given CSV files generated using file_acceleration().
file_en_velocity	Computes en_velocity() for given CSV files generated using file_velocity().
file_velocity	Computes velocity for CSV file generated by create_csv(), using velocity_x() and velocity_y().
file_video_index	Creates a video index using video_index() for a given directory and save it as a CSV file.
plot_frontal	Plot a heatmap of points extracted from *OpenPose* data using create_csv().
plot_timeseries	Plot a time series (velocity or acceleration), derived from *OpenPose* data using *OpenPoseR*.
velocity_x	Compute velocity for points on x-axis for given data frame in *OpenPoseR* format.
velocity_y	Compute velocity for points on y-axis for given data frame in *OpenPoseR* format.
video_index	Create an index of video files and their properties (length, frame rate, etc.) in a given directory.

*Notice that for all of the software’s core functionality, so-called wrapper functions with the prefix “file_” make it possible to directly work with files as input and output arguments, thereby making it easier to handle data derived from large sets of video clips.*

### Installation and Prerequisites

*OpenPoseR* was created using a current version of *R* (3.1 or newer; R Core Team, 2019). In addition, we recommend using a current version of *RStudio* (RStudio Team, 2020). Upon installation, *OpenPoseR* will automatically resolve its dependencies and install additional packages for plotting and reading video files which it requires to run in case they are missing. Notice that *OpenPoseR* provides a means of analyzing motion-tracking data generated by *OpenPose* (Cao et al., 2017, 2019), which is freely available for download.^2^ You will need to install and run *OpenPose* on your system or a compute cluster to fit body-pose models (Figure 1B) which can then be analyzed with *OpenPoseR*. Accordingly, an installation of *OpenPose* is not required on the same machine on which you use *OpenPoseR*, meaning that you can, for example, fit body-pose models on a central compute cluster with more powerful GPUs and analyze the output of *OpenPose* locally on your workstation or laptop. A number of different so-called “cloud computing” companies now also offer options to purchase GPU computing time in case such hardware is not available locally. *OpenPoseR* can be used to analyze data from all three standard models that can be fit with *OpenPose* (“body25,” “face,” or “hand”), whereas your choice of model of course depends on what exactly you want to control for. By default, we recommend fitting the “body25” model in the context of stimulus control. In the below example, we describe the analysis of data from the “body25” model, however, “face” and “hand” models can be fit and analyzed in the same manner.

The most straight-forward way of installing *OpenPoseR* on your machine is to use the install_github() function provided by the *devtools* package:

install.packages(”devtools”)

devtools::install_github(”trettenbrein/OpenPoseR”)

This will download and install the latest version of OpenPoseR on your system. Alternatively, it is also possible to use *R*’s ability to manually install packages from a downloaded archive of the source code, however, we strongly recommend to directly install the package from GitHub.

## Example

Below we outline an example workflow for using *OpenPoseR* to analyze *OpenPose* motion-tracking data derived from an example video clip. Throughout, we will use the results of the analysis of a short video clip showing the German Sign Language sign for PSYCHOLOGY (Figure 1A) in order to demonstrate the capabilities of *OpenPoseR*. All of *OpenPoseR*’s functionality can either directly be called upon in *R* scripts for data loaded into the *R* workspace or, alternatively, may indirectly be accessed using wrapper function (prefixed “file_”) which make it easier to work with data derived from large sets of video files (see Table 1 for an overview). The present example will use these wrapper functions to demonstrate how *OpenPoseR* would be used to compute the velocity of the body-pose model for the PSYCHOLOGY video clip. In addition to this abridged example, an interactive and reproducible demonstration of the approach including code examples and example data is available as an *R Markdown* file from the project’s GitHub repository.^3^

### Convert OpenPose Data

*OpenPose* creates a JSON file with information about the fit body-pose model(s) for every frame of the input video. Consequently, an input video file with a duration of exactly 5 s and a rate of 25 frames per second will already generate 125 individual files. To make these data easier to handle, *OpenPoseR* combines the output for an entire video file into a single CSV file with a human-readable tabular data structure. Depending on the model (“body25,” “face,” or “hand”) that has been fit using *OpenPose* the CSV file generated by *OpenPoseR* will contain consecutive columns for the x and y position of the points in the body-pose model followed by the confidence value (ranging from 0 to 1) for every point in the model. Every consecutive row contains the data of a frame of the input video clip. This conversion into the *OpenPoseR* format can be achieved using the create_csv() function:

create_csv(“input/path/”, “file_name”, “output/path/”)

The function will create a CSV file using the JSON files stored in the specified input directory with the suffix of the body-pose model (e.g., “_body25”) in the given output directory. In addition, the raw motion-tracking data may be viewed by plotting a heatmap of the data using the plot_frontal() function.

### Clean Motion-Tracking Data

On rare occasions, the model fit using *OpenPose* may contain zero values for x, y, and c of a point, indicating that the model fit failed for this particular point for the given frame. This is rather unlikely when working with stimulus video recordings which have been produced in the well-lit setting of professional video-recording facilities, but may occur with materials recorded in less optimal conditions. Similarly, points with very low confidence values (e.g., <0.3) might reasonably be excluded from further analysis as these values indicate that *OpenPose* had trouble detecting a point in a particular frame. Including zero values and frames with very low confidence values could lead to a situation where points may appear to be “jumping around” from frame to frame due to incorrect x and y values, thereby misrepresenting the actual velocity or acceleration of the model in the video clip.

Accordingly, to increase the accuracy of our calculations the data of the body-pose model should be cleaned and thresholded before further processing. *OpenPoseR* provides the clean_data() function which will take care of this step by automatically imposing zero values as well as values below a pre-defined cutoff using the mean of the previous and consecutive frames. By using file_clean() we can run this function for a large set of files:

file_clean(”path/to/file.csv”, cutoff=0.3, overwrite=FALSE)

This will create a file called “filename_model_cleaned.csv” because the argument “overwrite” was set to FALSE upon calling the function, thereby preventing OpenPoseR from overwriting the input CSV file with the cleaned data. Using the “cutoff” argument, the threshold for the confidence value that a point has to surpass can be adjusted. If everything went well, the function will return TRUE.

### Compute Velocity

After having successfully converted and cleaned the date, we can now compute the velocity of the different points of the body-pose model using *OpenPoseR*’s file_velocity() function. As already mentioned above, all functions prefixed with “file_” provide wrappers for *OpenPoseR* functions which make it possible to directly pass a filename to the function (see Table 1). In this case, file_velocity() will compute the velocity of a point on either the x- or y-axis using the velocity_x() and velocity_y() functions. Both functions compute the velocity of a point on either the x- or y-axis according to the following formula:

$$\frac{p_{t}-p_{t-1}}{t-\left(t-1\right)}$$

By using the file_velocity() function we can disregard these details regarding the computations being carried out and simply pass to the function the filename or a path to a CSV file in the *OpenPoseR* format as specified above in section “Clean Motion-Tracking Data”:

file_velocity(”path/to/file_model_cleaned.csv”)

Notice that if your video was recorded with more or less than 25 frames per second, you will have to specify this when calling the function by setting the argument “fps” to the desired value. Output files will be created automatically and carry either the suffix “_velocity_x” or “_velocity_y.” If everything went well, the function will return TRUE.

### Compute Euclidean Norm of Sums of Velocity Vectors

Given that our goal is to capture the total amount of bodily motion between frames in a single value, our final step in this analysis will be to compute the Euclidean norm of sums of velocity vectors (Figure 1C). In *OpenPoseR*, this is implemented in the en_velocity() function which takes the velocity vectors of the x- and y-axis as its input. The Euclidean norm of sums for velocity as the given motion feature then is computed using the following formula:

$$M\,F_{1}=||\sum_{i=1}^{N}v_{i}||$$

Again, we may disregard the details of the implementation by using the wrapper function file_en_velocity() which takes the filenames or path to the two CSV files created in the previous step as its input:

file_en_velocity(”path/to/file_model_cleaned_velocity_x.csv”,

”path/to/file_model_cleaned_velocity_y.csv”)

The output file will be created automatically and carries the suffix “_en_velocity.” If everything went well, the function will return TRUE.

### Plotting Results

Finally, the result of the previous computations can be visualized using basic plotting functions included in the package. *OpenPoseR* uses the functionality of *ggplot2* (Wickham, 2016) to provide some straightforward time series plots which can be created using the plot_timeseries() function:

example <- read.csv(”path/to/file_model_cleaned_en_velocity.csv”,

sep=””)”

plot_timeseries(example)

This will create the simple plot in Figure 1D which provides an illustration of how we have quantified the total amount of bodily motion occurring in the input video clip of the German Sign Language Sign PSYCHOLOGY. The sign is articulated with an initial large movement of the signer’s dominant right hand to the chest which is followed by a small hand movement in the same place on the chest, which is followed by another large movement of the hand into its original position. The onset and offset of this movement is clearly visible in the form of peaks in the timeseries in Figure 1D, thereby indicating that we succeeded in capturing the bodily motion occurring in the example video clip.

## Discussion and Outlook

The method and workflow presented here provides researchers in sign language and gesture studies with a straightforward and reproducible means for using body-pose estimation data derived from *OpenPose* for controlling their video stimuli. By quantifying the bodily movements of the actor in a video clip in terms of velocity or acceleration of a body-pose model it becomes possible to control for potential differences in these movement patterns, for example, across the different conditions of an experiment. In addition, this approach has already been successfully used to automatically detect the onset and offset for a large set of signs from German Sign Language (Trettenbrein et al., in press). *OpenPoseR*’s core functionality of computing measures of velocity and acceleration for *OpenPose* data is furthermore supplemented by functions for generating basic plots as well as an easy-to-use way of extracting metadata (e.g., duration, framerate, etc.) from large sets of video files. With its integration into the larger R environment, *OpenPoseR* supports reproducible workflows and enables seamless interaction with other packages in order to subject the motion-tracking results to further statistical analysis.

*OpenPoseR* was developed to assist sign language and gesture researchers with stimulus control in experiments that present participants with video recordings of an actor signing or gesturing, by reconstructing motion parameters of the actor using the data of body-pose models fit with *OpenPose*. However, we believe that the package’s functionality may also be useful for other domains of sign language and gesture research, especially as it can be continuously expanded due to the open-source nature of the project. For example, given that unlike other methods (e.g., Ramseyer, 2020) *OpenPose* does not require a static camera position, is sensitive to the actual body pose of the actor in the video, and it not biased by other motion or changes in the background, the method and workflow described here has the potential to enable the use of naturalistic stimuli in sign language research, similar to the increasing popularity of naturalistic stimuli in research on spoken language (Hamilton and Huth, 2020). Similarly, we believe that the functionality provided by *OpenPoseR* may prove useful in the automated analysis of large-scale *OpenPose* data derived from sign language corpora (e.g., Schulder and Hanke, 2019).

## Data Availability Statement

Publicly available datasets were analyzed in this study. This data can be found here: https://github.com/trettenbrein/OpenPoseR.

## Author Contributions

PT wrote *R* code and curated online resources. EZ supervised the project. Both authors conceptualized the functionality of the package, implemented algorithms, contributed to the article, and approved the submitted version.

## Conflict of Interest

The authors declare that the research was conducted in the absence of any commercial or financial relationships that could be construed as a potential conflict of interest.

## Footnotes

1 https://github.com/trettenbrein/OpenPoseR

2 https://github.com/CMU-Perceptual-Computing-Lab/openpose

3 https://github.com/trettenbrein/OpenPoseR/blob/master/demo.zip
